# A Prospective Longitudinal Assessment of Medical Records for Diagnostic Substitution among Subjects Diagnosed with a Pervasive Developmental Disorder in the United States

**DOI:** 10.3389/fped.2015.00085

**Published:** 2015-10-12

**Authors:** David A. Geier, Janet K. Kern, Brian S. Hooker, Lisa K. Sykes, Mark R. Geier

**Affiliations:** ^1^The Institute of Chronic Illnesses, Inc, Silver Spring, MD, USA; ^2^Simpson University, Redding, CA, USA; ^3^CoMeD, Inc, Silver Spring, MD, USA

**Keywords:** Asperger’s disorder, autism, cerebral palsy, diagnostic substitution, mental retardation, birth parameters

## Abstract

**Background:**

Previously, investigators suggested that diagnostic substitution from other diagnoses, e.g., mental retardation (MR) and/or cerebral palsy (CP) to pervasive developmental disorder (PDD) is a driving factor behind increases in autism. This study evaluated potential diagnostic substitution among subjects diagnosed with PDD vs. MR or CP by examining birth characteristic overlap.

**Methods:**

SAS^®^ and StatsDirect software examined medical records for subjects within the Vaccine Safety Datalink database who were Health Maintenance Organization-enrolled from birth until diagnosed with an International Classification of Disease, 9th revision (ICD-9) outcome of PDD (299.xx, *n* = 84), CP (343.xx, *n* = 300), or MR (317.xx, 318.xx, or 319.xx, *n* = 51).

**Results:**

Subjects with PDD had significantly (*p* < 0.01) increased: male/female ratio (PDD = 5.5 vs. CP = 1.5 or MR = 1.3), mean age of initial diagnosis in years (PDD = 3.13 vs. CP = 1.09 or MR = 1.62), mean gestational age in weeks at birth (PDD = 38.73 vs. CP = 36.20 or MR = 34.84), mean birth weight in grams (PDD = 3,368 vs. CP = 2,767 or MR = 2,406), and mean Appearance-Pulse-Grimace-Activity-Respiration scores at 1 min (PDD = 7.82 vs. CP = 6.37 or MR = 6.76) and 5 min (PDD = 8.77 vs. CP = 7.92 or MR = 8.04), as compared to subjects diagnosed with CP or MR.

**Conclusion:**

This study suggests diagnostic substitution cannot fully explain increased PDD prevalence during the 1990s within the United States.

## Introduction

Pervasive developmental disorders (PDD) are a diagnostic category characterized by delays in the development of socialization and communication skills (1). Although the typical onset of the symptoms associated with a PDD diagnosis occurs before a child is 3 years of age, parents may notice symptoms in early infancy (2). Problems with language; problems in relating to people, objects, and events; playing with toys and other objects in unusual ways; changes in routine or familiar surroundings being difficult; and body movements or behavior patterns that are repetitive are among the symptoms of a PDD diagnosis (3). Autism is the most common and best studied PDD. Asperger’s Syndrome, Childhood Disintegrative Disorder, and Rett’s Syndrome are other types of PDD diagnoses (3). Abilities, intelligence, and behaviors vary widely among those diagnosed with a PDD. Among those with a PDD diagnosis, some have no spoken language, while limited phrases or conversation abilities are present in some, and relatively normal language development is present in others. Limited sociability and repetitive play is often evident. Atypical sensory responses to loud noises and lights are also common (3). Gastrointestinal disturbances (48%), incontinence (57%), sleep problems (57%), eating disorders (94%), anxiety/fear (74%), behavioral problems (89%), and obsessive-compulsive behaviors (92%) are commonly reported health, physical, and behavior symptoms among individuals diagnosed with a PDD (4).

It is apparent that PDD prevalence has increased 20- to 30-fold since studies conducted in the 1960s/1970s. In the 1960s/1970s, it was estimated that 1 in 2,500 children (0.04%) were diagnosed with a PDD (5), but by the 2000s prevalence estimates ranged from 1 to about 2% of all children without any correction for under ascertainment (6, 7).

As described previously by investigators (8), the Autism and Developmental Disabilities Monitoring (ADDM) Network is an active surveillance system providing estimates of the prevalence of autism spectrum disorder (ASD) among children 8 years-old in specific areas of the United States (US) without any correction for under ascertainment. The earliest reports from the ADDM Network provided estimates of ASD prevalence among children for the 2000 and 2002 surveillance years. Data from 2000 and 2002 surveillance years indicated that the ASD prevalence was 6.7 per 1,000 in 2000 and 6.6 per 1,000 in 2002, or approximately 1.0 in every 150 children (0.67%). It was reported in subsequent surveillance years that the estimated ASD prevalence rose to 8.0 per 1,000 in 2004 and 9.0 per 1,000 in 2006, or about 1.0 in every 110 children in 2006 (0.9%). It was latter reported that for the 2008 surveillance year the prevalence of ASD increased to 11.3 per 1,000 children, or one in 88 children (1.13%). Finally, it was reported that for the 2010 surveillance year the ASD prevalence rose to 14.7 per 1,000 children, or one in 68 children (1.47%).

Since almost all of the PDD cases are ASD cases, there have been significant increases in estimated PDD prevalence in the US. Despite these increases in the size and the magnitude of ASD, some investigators have continued to suggest extrinsic factors such as changes in diagnostic practice account for much of the rise in ASD prevalence (9–11). Specifically, these investigators have suggested that diagnostic substitution from other diagnosis such as mental retardation (MR) and/or cerebral palsy (CP) to PDD is the driving factor behind the increases in PDD. As a result, a longitudinal hypothesis-testing epidemiological study was commenced to evaluate the potential for diagnostic substitution among children diagnosed by healthcare providers with a PDD in comparison to being diagnosed with MR or CP by evaluating automated medical records within the Vaccine Safety Datalink (VSD) database for the degree of overlap of various birth parameters associated with each of the diagnostic outcomes examined.

## Materials and Methods

The Institutional Review Board (IRB) of Kaiser Permanente North-West (KPNW), and the IRB of Kaiser Permanente Northern California (KPNC), as well as the US Centers for Disease Control and Prevention (CDC) approved the study protocol. The Research Data Center of the National Center for Health Statistics in Hyattsville, MD, US served as the secure location where the data for the present study was analyzed. CDC or Kaiser Permanente does not necessarily agree with the views expressed in this study.

As previously described, medical event information, specific vaccine history, and selected demographic information from the computerized databases of several Health Maintenance Organizations (HMOs) is linked in the VSD project (12–14). The National Immunization Program (NIP) created the VSD project in 1991. Children with non-missing dates of birth and gender, continuously enrolled within a VSD-participating HMO from birth, and had HMO birth files with no missing values were examined in this study.

### Population at Risk

SAS^®^ software was utilized to examine a cohort of over one million infants enrolled in the VSD project (updated through the end of 2000) from KPNW, Kaiser Permanente Colorado (KPC), and KPNC.

### Determining Subjects with Outcomes

The first instance of International Classification of Disease, 9th revision (ICD-9) healthcare provider diagnosed PDD (299.xx), CP (343.xx), or MR (317.xx or 318.xx or 319.xx) were identified from the outcome files (inpatient and outpatient diagnoses) in the VSD database. Given that a child might be diagnosed multiple times with the same diagnosis, only the child’s initial diagnosis was evaluated. It is also possible for subjects to be counted in more than one outcome category, since subjects with multiple different diagnoses were included in the present study. For the outcomes examined, Table 1 summarizes their overall demographics.

**Table 1:** **A summary of the various types of outcomes examined in the present study**.

Group examined^a^ (ICD-9 code)	Number of males	Number of females	Male/female ratio	Birth years	Mean age of initial diagnosis **±** std (95% CI)
Pervasive developmental disorder, *n* = 84 (299.xx)	71	13	5.5^b^	1995–1999	3.13 ± 0.81^c^ (2.96–3.30)
Cerebral palsy, *n* = 300 (343.xx)	179	121	1.5	1995–2000	1.09 ± 0.81 (1–1.18)
Mental retardation, *n* = 51 (317.xx or 318.xx or 319.xx)	29	22	1.3	1995–2000	1.62 ± 1.11 (1.31–1.92)

*^a^Subjects examined were continuously HMO-enrolled from birth until their initial diagnosis of the outcome examined*.

*^b^There was a significantly increased male/female ratio among subjects diagnosed with a pervasive developmental disorder in comparison to cerebral palsy (  0.0001) or mental retardation (  0.001) using the Fisher’s exact test statistic*.

*^c^The mean age of initial diagnosis with significantly longer among subjects diagnosed with a pervasive developmental disorder in comparison to cerebral palsy [()  20.4, df  133,   0.0001] or mental retardation [()  8.4, df  82,   0.0001] using the test statistic*.

### Determining Birth Demographics

Among the outcomes examined, the HMO birth file for each child was examined to determine each child’s gestational age in weeks at birth, birth weight in grams, and Appearance-Pulse-Grimace-Activity-Respiration (APGAR) score at 1 and 5 min. In addition, the number of males and females and the age of each child’s initial diagnosis were determined for each outcome examined.

### Statistical Analyzes

The StatsDirect (version 3.0.150) software was utilized for statistical analyzes, and a two-sided *p*-value < 0.05 was considered statistically significant. The Fisher’s exact test statistic was utilized to compare the male/female ratio among subjects diagnosed with a PDD in comparison to subjects diagnosed with CP or MR. The *t* test statistic (assuming unequal variances) was utilized to compare the birth demographic parameters of gestational age at birth, birth weight, APGAR scores at 1 and 5 min, and mean age of the initial diagnosis for each outcome studied. The null hypothesis was that for each comparison examined there would be no difference among the subjects diagnosed with a PDD in comparison to subjects diagnosed with CP or MR.

## Results

Overall, the present assessment of the VSD database for children without any missing information in their HMO birth file identified 84 subjects diagnosed with PDD (born between 1995 and 1999), 300 subjects diagnosed with CP (born between 1995 and 2000), and 51 subjects diagnosed with MR (born between 1995 and 2000) who were born into the system and continuously enrolled until their diagnosis. Table 1 reports the male/female distribution, birth years, and mean age of initial diagnoses for each of the outcomes examined in this study. Subjects diagnosed with a PDD were found to have had a significantly increased male/female ratio (male/female ratio = 5.5) in comparison to subjects diagnosed with CP (male/female ratio = 1.5, *p* < 0.0001) or MR (male/female ratio = 1.3, *p* < 0.001). In addition, the mean age of initial diagnosis in years for subjects who were diagnosed with a PDD was significantly older (3.13 ± 0.81) than the mean age of initial diagnosis for subjects who were diagnosed with CP (1.09 ± 0.81, *p* < 0.0001) or MR (1.62 ± 1.11, *p* < 0.0001).

Table 2 summarizes the results found for the various types of birth measurements examined among subjects diagnosed with the various outcomes examined in the present study. Notably, the mean gestational age in weeks at birth among subjects diagnosed with a PDD (38.73 ± 2.48) was significantly increased in comparison to the mean gestational age at in weeks at birth among subjects diagnosed with CP (36.20 ± 2.47, *p* < 0.0001) or MR (34.84 ± 2.48, *p* < 0.0001). In addition, the mean birth weight in grams among subjects diagnosed with a PDD (3,368 ± 749) was significantly greater in comparison to subjects diagnosed with CP (2,767 ± 735, *p* < 0.0001) or MR (2,406 ± 733, *p* < 0.0001). Finally, APGAR scores at 1 and 5 min among subjects diagnosed with a PDD were significantly increased (7.82 ± 1.78 and 8.77 ± 0.75) in comparison to the corresponding APGAR scores for subjects diagnosed with CP (6.37 ± 1.73 and 7.92 ± 0.74, *p* < 0.0001) or MR (6.76 ± 1.75 and 8.04 ± 0.73 *p* < 0.0005).

**Table 2:** **A summary of the various types of birth measurements examined among the outcomes studied**.

Group examined^a^ (ICD-9 code)	Mean gestational age at birth in weeks **±** SD (95% CI)	Mean birth weight in grams **±** SD (95% CI)	Mean APGAR at 1 min **±** SD (95% CI)	Mean APGAR at 5 min **±** SD (95% CI)
Pervasive developmental disorder, *n* = 84 (299.xx)	38.73 ± 2.48^b^ (38.19–39.26)	3,368 ± 749^c^ (3,208–3,528)	7.82 ± 1.78^d^ (7.44–8.20)	8.77 ± 0.75^e^ (8.61–8.93)
Cerebral palsy, *n* = 300 (343.xx)	36.20 ± 2.47 (35.92–36.49)	2,767 ± 735 (2,683–2,850)	6.37 ± 1.73 (6.17–6.57)	7.92 ± 0.74 (7.84–8.00)
Mental retardation, *n* = 51 (317.xx or 318.xx or 319.xx)	34.84 ± 2.48 (34.16–35.52)	2,406 ± 733 (2,206–2,608)	6.76 ± 1.75 (6.28–7.24)	8.04 ± 0.73 (7.84–8.24)

*95% CI, 95% confidence interval; APGAR, appearance-pulse-grimace-activity-respiration; ICD-9, international classification of disease, 9th revision*.

*^a^Subjects examined were continuously HMO-enrolled birth until their initial diagnosis of the outcome examined*.

*^b^There were significantly increased mean gestational ages among subjects diagnosed with a pervasive developmental disorder in comparison to cerebral palsy [()  8.27, df  133,   0.0001] or mental retardation [()  8.84, df  106,   0.0001] using the test statistic*.

*^c^There were significantly increased mean birth weights among subjects diagnosed with a pervasive developmental disorder in comparison to cerebral palsy [()  6.53, df  131,   0.0001] or mental retardation [()  7.33, df  107,   0.0001] using the test statistic*.

*^d^There were significantly increased mean APGAR scores at 1 min among subjects diagnosed with a pervasive developmental disorder in comparison to cerebral palsy [()  6.64, df  130,   0.0001] or mental retardation [()  3.39, df  107,   0.005] using the test statistic*.

*^e^There were significantly increased mean APGAR scores at 5 min among subjects diagnosed with a pervasive developmental disorder in comparison to cerebral palsy [()  9.21, df  132,   0.0001] or mental retardation [()  5.57, df  108,   0.0001] using the test statistic*.

## Discussion

The results of the present hypothesis-testing prospective, longitudinal, case study revealed that the subjects diagnosed by healthcare providers with a PDD in comparison to MR or CP within the VSD database were different populations. There were highly statistically significant differences in the male/female ratio, mean age of initial diagnosis, gestational age at birth, mean birth weight, and APGAR scores at 1 and 5 min when comparing subjects diagnosed with a PDD to those subjects diagnosed with CP or MR. As a consequence, the results observed refute the hypothesis that diagnostic substitution can significantly explain the observed increase in PDD prevalence in the US during the 1990s.

In comparing our results with previous investigations that purportedly suggested diagnostic substitution as a factor contributing to the increasing prevalence of PDD in the US during the 1990s, the present study used a much more robust method of analysis to confirm that the population of subjects diagnosed with a PDD was significantly different than the population of subjects diagnosed with CP or MR. This more robust method of analysis examined data on specific features which described subjects at birth (independent of their eventual diagnosis), and followed these children on a continuous, prospective longitudinal basis within their respective HMOs until they were diagnosed with the outcomes of interest.

By contrast, previous studies purportedly suggesting diagnostic substitution as a factor in or an explanation for increases in PDD prevalence have examined only population-based trends in diagnoses (i.e., PDD prevalence rates increased as MR prevalence rates decreased). For example, investigators conducted a population-based study of children born in California from 1987 to 1994 who had an autism diagnosis (10). Trends in the prevalence of a MR diagnosis without an autism diagnosis were also investigated to evaluate the role of diagnostic substitution. The prevalence of autism increased from 5.8 to 14.9 per 10,000 subjects, for an absolute change of 9.1 per 10,000 subjects, during the study period examined. The prevalence of a MR diagnosis without autism decreased from 28.8 to 19.5 per 10,000 subjects, for an absolute change of 9.3 per 10,000 subjects, during the same period. These investigators concluded that their data suggest that the observed increase in autism could be accounted for by the change in MR diagnoses.

Similarly, in evaluating the potential for diagnostic substitution between PDD and MR diagnoses at the population level, other investigators reported that the prevalence of a MR diagnosis in special education declined by 2.8 per 1,000 subjects from 1994 to 2003, whereas autism prevalence increased by 2.6 per 1,000 subjects (9). These investigators concluded that the total decline in MR diagnosis could have offset the total increase in autism prevalence to almost 1-for-1.

Investigators also attempted to ascertain whether diagnostic substitution was responsible for the increased prevalence of autism in the state of California (11). These investigators estimated that 26.4% (95% confidence interval = 16.25–36.48%) of the increased autism caseload in California was uniquely associated with diagnostic change through a single pathway – individuals previously diagnosed with MR. As a consequence, their estimate of diagnostic change could account for no more than a 26.4% increase in autism diagnoses.

In addition, another study published in 2009 (15), found that diagnostic changes could only partially account for the increases in the prevalence of those children receiving an autism diagnosis. These investigators found only one-third of the increase in the total rate of autism diagnosis could be explained by changes in diagnostic criteria, inclusion of milder cases, and earlier age at diagnosis.

In a more recent study conducted in California, investigators examined key social factors (e.g., diagnostic dynamics, community resources, and demographics) that were considered to have contributed to increased autism prevalence (16). Even including these social factors, the researchers determined that only about half of the increase in autism prevalence could be explained. They stated that the changing patterns of identification and treatment could not completely account for the other percentage increase in the prevalence of an autism diagnosis.

All told, the aforementioned studies have significant limitations in their ability to explain the significant increases in the prevalence of PDD in the US during the 1990s. For example, some of the aforementioned studies attempted to explain increased trends in the prevalence of PDD diagnoses by reporting that increased trends in the prevalence of PDD diagnoses correlated with decreased trends in the prevalence of MR diagnoses. Despite attempting to make a connection between these two phenomena, the investigators did nothing in their studies to attempt to evaluate clinical characteristics such as gender ratio, age of initial diagnosis, birth weight, APGAR scores, or gestational age at birth among the actual diagnosed subjects to determine, what if any, overlap existed in clinical characteristics at the individual level to explain the phenomena observed. In addition, some of the aforementioned studies failed to consider that there are several different diagnoses that compose the PDD spectrum, and as a consequence, they may have failed to report the true extent of the increase in the prevalence of PDD in the US during the 1990s. Another significant limitation of some of the aforementioned studies is that they examined children born within a limited range for birth years (i.e., examining children only born from 1987 to 1994), and as a consequence, the studies may not have been able to fully report on the increased prevalence of PDD in the US during the 1990s. Also, some of the other aforementioned studies reported a limited amount of the increases in the rates of autism diagnoses observed could be explained by changes in diagnostic criteria, inclusion of milder cases, and earlier age at diagnosis. Even when the investigators in some of the aforementioned studies attempted to evaluate the potential impact of key social factors such as diagnostic dynamic, community resources, and demographics, these changes could only explain a fraction of the increased prevalence of PDD.

Considering the findings reported in the present study, the results observed are consistent with the findings of other investigators from the CDC who examined birth weight and gestational age characteristics among children diagnosed with autism in comparison to those diagnosed with CP or MR (17). Consistent with the results obtained in the present study, those investigators observed that the prevalence of children with an autism diagnosis in low birth weight or preterm children was markedly lower than those diagnosed with MR or CP.

### Strengths/Limitations

An important potential strength of this study was that a retrospective assessment of the VSD database was undertaken to evaluate prospectively generated medical records of patients. Indeed, all of the subjects examined in this study, were enrolled continuously in one of the VSD-participating HMOs from birth until their respective diagnoses. As a consequence, factors associated with enrollment (i.e., adjustment for potential independent variables between the different outcomes was not necessary because enrollment was from birth to the child’s diagnoses) or healthcare-seeking behavior (i.e., adjustment for potential access/availability of healthcare was continuous for all the outcomes examined) were minimized in this study. Further, since, the VSD data records analyzed were collected as part of the a subject’s routine healthcare (i.e., many years before a subject was diagnosed with the outcomes under study), it is reasonable to hypothesize the healthcare providers were not influenced in reporting the various variables examined between subjects diagnosed with a PDD in comparison to those diagnosed with MR or CP.

Another potential strength of this study was that consistent diagnostic coding was used at the HMOs examined (i.e., ICD-9 coding was consistently employed). As a consequence, differences in diagnostic coding between various healthcare professionals were minimized. In addition, since all of the subjects examined in this study were born and diagnosed with the various outcomes after 1994, all were diagnosed under the Diagnostic and Statistical Manual of Mental Disorders, 4th Edition (DSM-IV) (18), so that differences in the DSM criteria remained consistent for the outcomes examined.

However, there a number of potential limitations to this study. Unknown biases, confounders, or statistical chance may account for the results observed. These potential limitations seem unlikely given the consistent direction, high degree of statistical significance, and limited number of statistical tests performed in this study. Still, future studies should examine other databases for consistency with the observations made in this study.

Another potential limitation of this study is that subjects could have received more than one of the outcomes under study, and as such, their data would have been included in each of the outcomes examined. This potential overlap of individuals between outcomes examined would bias the results observed toward the null hypothesis (i.e., minimizing the differences for the various parameters examined between the various outcomes examined), but despite this limitation, there were consistent and highly significant differences between subjects diagnosed with a PDD in comparison to those diagnosed with MR or CP.

Potential additional limitations of this study include that some subjects examined by their healthcare providers may not have been aware of more subtle neurological dysfunction, some subjects may have been misdiagnosed by their healthcare providers, or some of the variables examined may have been incorrectly recorded. A significant impact on the results observed in this study should not have occurred from the aforementioned limitations because it is unclear how differential application would have occurred to result in the phenomena observed. In addition, it is important to realize that data misclassification would tend to bias any results toward the null hypothesis.

A still further potential limitation of this study is that subjects examined within the VSD database may be different than the general US population, and, as a consequence, the findings made may not be necessarily generalizable to the general US population. This potential limitation was recently investigated by researchers who determined that the VSD population is representative of the general US population on several key demographic and socioeconomic attributes (19). These researchers also reported that the VSD population is large enough to ensure an adequate representative sample of the general US population.

## Conclusion

The results of the present study revealed that subjects diagnosed by healthcare providers with a PDD in comparison to those diagnosed with MR or CP, within the VSD database were significantly different populations, and, as a result, these differences suggest that diagnostic substitution cannot fully explain the increase in diagnosed PDD during the 1990s within the US. The observations made in the present study call into question the sufficiency of previous studies, which have sought to evaluate diagnostic substitution as an explanation for the increase in PDD during the 1990s in the US by comparing only prevalence changes in diagnosed PDD to prevalence changes in diagnosed MR or CP; such an evaluation would seem too superficial to yield any valid results without additional clinical comparisons of the populations in question. The observations made in the present study also provide critical insights for healthcare providers attempting to identify important and distinguishing clinical features associated with a diagnosis of PDD in comparison to a diagnosis of MR or CP. Among the important clinical features associated with those diagnosed with a PDD observed in the present study were: elevated male/female ratio (males significantly more likely than female to be effected); delayed mean age of initial diagnosis (mean age of initial diagnosis between 2 and 4 years-old); and the lack of significant problems at birth in comparison to those with a MR or CP diagnosis. By contrast, among the important clinical features observed in the present study and associated with those diagnosed with MR or CP were: roughly equal male/female ratio (males roughly equally likely with female to be effected); early mean age of initial diagnosis (mean age of initial diagnosis within the first 2 years of life); and with significant problems at birth in comparison to those with a PDD diagnosis.

## Author Contributions

DG and MG conceived the study, conducted the research, involved in the interpretation of the data, and drafting and revising of the manuscript. JK was involved in the interpretation of the data and drafting and revising the manuscript. BH was involved in computer programing, conducted the research, and drafting and revising the manuscript. LS was involved in the drafting and revising the manuscript.

## Conflict of Interest Statement

The authors declare that the research was conducted in the absence of any commercial or financial relationships that could be construed as a potential conflict of interest.

## Abbreviations

ADDM, autism and developmental disabilities monitoring; APGAR, Appearance-Pulse-Grimace-Activity-Respiration; ASD, autism spectrum disorder; CDC, Centers for Disease Control and Prevention; CP, cerebral palsy; DSM-IV, Diagnostic and Statistical Manual of Mental Disorders, 4th Edition; HMOs, Health Maintenance Organizations; ICD-9, International Classification of Disease, 9th revision; IRB, Institutional Review Board; KPC, Kaiser Permanente Colorado; KPNC, Kaiser Permanente Northern California; KPNW, Kaiser Permanente North-West; MR, mental retardation; NIP: National Immunization Program; PDD, pervasive developmental disorder; US, United States; VSD, vaccine safety datalink.
